# The Efficacy of Voluntary Self-Exclusions in Reducing Gambling Among a Real-World Sample of British Online Casino Players

**DOI:** 10.1007/s10899-023-10198-y

**Published:** 2023-03-25

**Authors:** Niklas Hopfgartner, Michael Auer, Denis Helic, Mark D. Griffiths

**Affiliations:** 1 Institute of Interactive Systems and Data Science, Graz University of Technology, Sandgasse 36/III, Graz, 8010 Austria; 2 neccton GmbH, Davidgasse 5, Müllendorf, 7052 Austria; 3 International Gaming Research Unit, Psychology Department, Nottingham Trent University, 50 Shakespeare Street, Nottingham, NG1 4FQ UK

**Keywords:** Gambling, Responsible gambling, Responsible gambling tools, Problem gambling, Voluntary self-exclusion, Play break

## Abstract

Online gambling is a socially acceptable means of entertainment, but it can also have a negative impact on many areas of life and lead to problem gambling for a minority of individuals. In recent years, gambling operators have increasingly implemented responsible gambling tools to help at-risk gamblers control and limit their gambling. One such tool is voluntary self-exclusion (VSE), where gamblers can exclude themselves from the gambling platform for a self-selected period of time. Despite the widespread use of VSE, there are few published studies on the efficacy of VSE among online gamblers and none on whether (and what type of) gamblers return to gambling after self-exclusion and how VSE affects their wagering if they return. Using a secondary dataset, the present study empirically analyzed a real-world sample of 3,203 British online casino players who opted for a VSE between January 2021 and August 2022. Analysis showed that most players who took a short-term VSE (up to 38 days) started gambling again on the platform after their self-exclusion ended, while players who opted for long-term self-exclusion (more than 90 days) did not start gambling again on the platform. A return to the gambling platform after VSE was positively associated with (i) a shorter duration of the self-exclusion, (ii) being female, (iii) gambling on more days, (iv) placing more bets, (v) playing fewer type of games, and (vi) having a lower average number of deposits per day. Players who returned from VSE did not change their wagering compared to a matched control group. These results suggest that short-term VSE may not be as effective as long-term VSE in reducing gambling. Overall, the present findings suggest that gamblers returning from VSE should be closely monitored, especially if the reason for self-exclusion is related to problem gambling.

## Introduction

Online gambling involves the wagering of money on the internet via Wi-Fi-enabled electronic devices (e.g., desktop computers, laptops, smartphones, tablets, etc.). Recent data from the UK Gambling Commission reported an 8% increase in the total number of bets and a 13% increase in monthly active accounts, indicating continued popularity of online gambling in Great Britain (Gambling Commission, 2023). Although online gambling is a safe means of entertainment for most individuals, an excessive engagement in such an activity can increase the risk of developing a gambling disorder, which in turn can lead to negative consequences, such as gambling-related crimes, substance misuse, and personality disorders, among others (Adolphe et al., 2019; Petry et al., 2005).

Mora-Salgueiro et al. (2021) systematically reviewed studies that included data on problematic online gambling as well as sociodemographic and comorbid variables related to it. Estimates for adults who could be classified as problem gamblers ranged from 2.7% to 20.3%. Prevalence rates of at-risk and problem gambling among adolescents ranged from 5.7% to 57.5%. Being single and being male have been found to be indicative for online gambling disorder in a number of the studies reviewed. Hing et al. (2022) compared land-based-only gamblers (LBOGs), online-only gamblers (OOGs), and mixed-mode gamblers (MMGs), based on a 2019 Australian national telephone survey (N = 15,000). MMGs had the highest gambling involvement, gambling problems and gambling-related harm. Furthermore, gambling on the internet (OOGs and MMGs combined) was associated with higher problem gambling severity than land-based-only gambling (LBOGs). Similar findings were also reported in a large-scale nationally representative study (N = 7,756) in Great Britain (Wardle et al., 2011). Díaz and Pérez (2021) studied the association between online gambling participation, the intensity of participation, and the risk of developing gambling-related harm using data from the prevalence study in Spain. They found that online gambling had a significant impact on the likelihood of developing a gambling disorder, which worsened with increased participation in online gambling.

### Responsible gambling

Hing et al. (2018) synthesized findings from a systematic literature review, website analysis, and online survey of 107 experts to investigate the definition of responsible consumption of gambling (RCG). They distilled the following principles underpinning RCG: (i) *affordability*: gambling within an individual’s affordable limits of time, money, and other resources, which may involve setting and adhering to pre-committed limits; (ii) *balance*: keeping gambling in balance with other activities, responsibilities, and priorities so that it does not compromise other aspects of the gambler’s life; (iii) *informed choice*: exercising informed choice over gambling which includes understanding the associated risks, not being influenced by erroneous gambling beliefs, and knowing the odds or the likelihood of losing and winning; (iv) *control*: staying in control of gambling through individuals self-regulating their own gambling and knowing when to stop; (v) *enjoyment*: enjoying the gambling experience and being motivated by gambling only for pleasure, entertainment, and fun rather than winning money; and (vi) *harm-free*: gambling in a way that avoids the development of gambling problems that comprises the absence of gambling-related harm to self and others.

### Voluntary self-exclusion

In order to support gamblers to engage in responsible gambling (RG) consumption, regulators mandate the gambling industry to provide various RG tools to inhibit the development of problem gambling and reduce associated harms. One such tool is the voluntary self-exclusion (VSE). Players can exclude from an online gambling site at any time and often for flexible periods of time. Often VSEs are valid across operators once a player is reported to a system such as *GAMSTOP* (McCormick & Cohen, 2007) in the UK or *Spelpaus* (Håkansson & Widinghoff, 2020) in Sweden. In a study of the 50 most advertised gambling operators worldwide, Catania and Griffiths (2021a) reported that most of them offered the possibility to set limits (98%) and to self-exclude (96%).

Several studies have utilized account-based behavioral player tracking data to evaluate VSEs. Some of these studies have argued that gamblers who used VSE were too different (e.g., in terms of their gambling activity prior to VSE) to be treated as a homogeneous group, and that problem gamblers may not yet be at the stage where they are willing to change their behavior. Therefore, VSE is not a reliable proxy measure for problem gambling (Griffiths & Auer, 2016; Catania & Griffiths, 2021b). However, some studies have analyzed behavioral markers associated with VSEs, and argued that markers such as frequent limit changes and short-term VSEs, use of multiple payment methods, frequent in-session deposits, or playing multiple types of games may indicate potentially problematic gambling behavior (Finkenwirth et al., 2021; Hopfgartner et al., 2023).

A recent study based on self-reported data from Swedish players (i.e., Håkansson & Widinghoff, 2020) reported that self-excluders were more likely to (i) be younger, (ii) be female, and (iii) play chance-based games and online poker. Moreover, VSE also remained strongly associated with past-year online casino gambling and gambling problems. Håkansson and Åkesson (2022) analyzed a sample of 85 Swedish treatment seekers and reported that 68% of self-excluded gamblers continued to gamble on unlicensed online gambling sites during their VSE. Caillon et al. (2019) investigated the efficacy of VSE among a sample of 60 French online gamblers and reported that a seven-day self-exclusion had no effect on money wagered or time spent gambling, but that ‘illusion of control’ and ‘perceived inability to stop gambling’ decreased significantly over the following two months. Luquiens et al. (2019) analyzed behavior of French online poker players after the expiration of a VSE and found a significant decrease in money and time spent over the following 12 months. However, the study found no significant effect on the amount of money spent among the gamblers that were the most heavily involved financially.

### The present study

As far as the present authors are aware, there is no previous research regarding the return rate of players who voluntarily self-excluded and the associated behavioral risk factors that might facilitate such a return, based on a real-world sample of online casino players. Furthermore, players who return from a VSE could potentially spend even more than before their VSE because Blaszczynski et al. (2015) argued that short breaks in play could increase craving and therefore lead to increased gambling. Therefore, the present study was designed to provide more insights into players’ real-word behavior after the VSEs expired by investigating the following research questions (RQs): (i) how many gamblers return after a VSE? (RQ1); (ii) what type of gambling profile (i.e., the type of gambler) is associated with returning after a VSE? (RQ2); and (iii) does the amount of money wagered change after a VSE? (RQ3). The authors believe that the answers to these RQs could assist in identifying players who might need further support after a VSE because in recent years, regulators have mandated gambling operators to monitor player behavior and intervene in cases of suspected risky and/or problematic gambling.

## Methods

The authors were given access to an anonymized secondary dataset from a British online casino operator which contained the gambling behavior of all active players between January 1, 2021 and August 5, 2022. More specifically, the data included daily aggregates for money wagered, lost, deposited, and withdrawn, as well as information about the type of games played and the payment methods used. The dataset also contained the start and end dates of every VSE event that occurred during that period.

### Study design

The authors only included individuals who placed at least one bet in the 30 days prior a VSE and aggregated their gambling behavior during that period. If gamblers returned after a VSE, the gambling behavior in the 30 days after they started to gamble again was also aggregated. To ensure that all players had at least 30 days to gamble, all players who self-excluded in less than 30 days after their registration were removed from the dataset. Furthermore, only players who returned from a VSE 30 days prior the last available day in the dataset (i.e., August 5, 2022), or had their self-exclusion end at least 30 days prior that day were considered. Finally, only the first VSE of each gambler was used to avoid a bias towards gamblers who frequently used short VSEs. The reason for choosing a 30-day time range was pragmatic because it ensured that all players had sufficient time to gamble both before and after the VSE.

For better comparability and to control for potential confounding effects, the authors also created a control group using a matched-pairs design. This means that for each player who opted for a VSE, a matched pair (i.e., a player with a similar gambling profile) was determined who gambled at the same time in a similar way but did not opt for a VSE. Out of all players, one player which was most similar was chosen. The following criteria were used to identify a set of similar non-self-excluders for each gambler who opted for a VSE:

- *Age*: The matched gamblers were required to be in the same age group. The various thresholds for the age groups were adopted from Wardle et al. (2011) where age was categorized into seven groups: <24 years, 24–34 years, 35–44 years, 45–54 years, 55–64 years, 65–74 years, and > 75 years.
- *Gender*: The matched gamblers were required to be the same gender.
- *Money wagered*: The matched gamblers were required to have been active on the day of the VSE, and they were required to have a similar amount of money wagered in the last 30 days prior to that event. More specifically, the money wagered was only allowed to deviate up to 10% in either direction.
- *Number of active days*: The matched gamblers were required to have a similar number of active gambling days (±3 days) in the 30 days prior the VSE.
- *Game type profile*: The matched gamblers were required to have played the same type of games in the 30 days prior the VSE. For example, if a player who opted for a VSE played slots, blackjack, and roulette, the matched gamblers were also required to have played the same type of games.

**Fig. 1:**
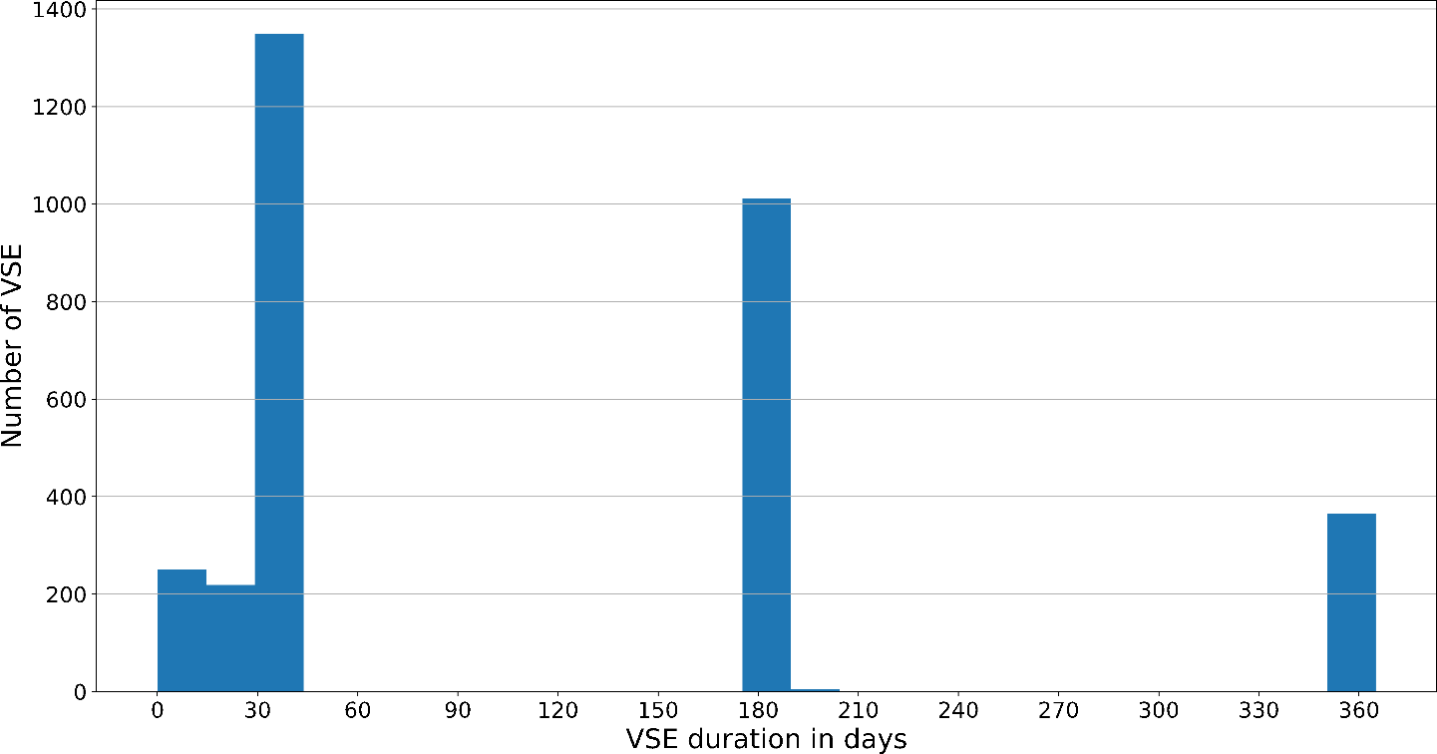
Distribution of the duration of voluntary self-exclusion (VSE) showing three typical durations (30 days, 180 days, and one year)

After applying the aforementioned criteria, the one gambler with the least difference with respect to amount of money wagered was selected if more than one matched pair per player remained. Finally, the present study also controlled for the duration of the VSE by categorizing gamblers into two groups. Figure 1 shows the distribution of the VSE duration, which indicates that players generally opt for either short-term VSEs (i.e., up to 38 days) or long-term VSEs ranging from 90 days to one year. Therefore, the threshold for distinguishing between short-term and long-term VSEs was set at 38 days, resulting in two almost identically sized groups in terms of the number of players.

### Participants

In total, there were 3,203 gamblers who opted for a VSE between January 2021 and August 2022 after accounting for the aforementioned requirements. The average age was 39.4 years, and 51.3% of the gamblers were male. Table 1 shows the descriptive statistics of gamblers who took a short-term or long-term VSE. The average duration for short-term VSEs was 27 days, and the average duration for long-term VSEs was 230 days. There was no significant difference in the age between gamblers who took a short-term or long-term VSE (Mann-Whitney U-test: *U* = 1,309,190, *p* = .051). However, the percentage of male gamblers who opted for a long-term VSE (55.5%) was significantly higher as compared to the percentage of male gamblers who opted for a short-term VSE (48.1% - chi-square test: *χ*^2^ = 17.14, *p* < .001). Moreover, gamblers who opted for a long-term VSE wagered significantly less money in the 30 days prior the VSE (*U* = 1,364,389, *p* < .001), and gambled on significantly less days (*U* = 1,434,421, *p* < .001) as compared to gamblers who opted for a short-term VSE.

**Table 1:** Descriptive statistics of gamblers who took a short-term VSE (up to 38 days) or long-term VSE (90 days to one year)

VSE duration	Number of gamblers	Mean duration of VSE (days)	Mean age (years)	Number of male gamblers	Median money waged 30 days prior VSE	Median number of active days
Short-term	1,820	27	39.8	875 (48.1%)	1,078	5
Long-term	1,383	230	38.9	767 (55.5%)	817	4
	3,203	115	39.4	51.3	964	4

### Statistical analysis

To evaluate how many players started to gamble again after a VSE (RQ1), the authors calculated the percentage of gamblers who returned after a VSE. As described previously, it was ensured that each player had at least 30 days to return after a VSE. For RQ2, the authors first tested whether gamblers with high stakes were more likely to return than gamblers with low stakes. Therefore, gamblers were divided into four equally sized groups according to how much money they had wagered in the 30 days prior to their VSE, and the percentage of gamblers who returned was calculated for each group. Second, a logistic regression was performed to identify other behavioral factors that were associated with a return to gambling after a VSE. The dependent variable indicated whether gamblers returned from a VSE, and behavioral variables derived from the player tracking data were used as independent variables. The list of behavioral variables after removing highly correlated variables using a variance inflation factor of smaller than 10 (James et al., 2013) is provided in Appendix 1. To evaluate whether gamblers increased or decreased the amount wagered after they returned following a VSE (RQ3), the previously described matched-pairs procedure was applied. For 1,413 out of the 3,203 gamblers (44%), a matched pair was found. If the VSE had no effect on gamblers who returned and started to gamble again, 50% of the gamblers would stake more than their matched pairs, and the other 50% would stake less than their matched pairs. Therefore, it was assumed that any deviation from this uniform distribution (i.e., equal to flipping a fair coin) would be caused by the VSE. Consequently, the difference between the observed percentage and the expected percentage (i.e., 50%) of gamblers who increased their stakes was statistically examined. To measure the actual strength of such an effect, a difference-in-differences regression was also applied to separate actual intervention effects from potential concurrent (e.g., seasonal) effects. To do so, the difference between the stakes before and after a VSE was calculated and compared to the difference in stakes in the same period for gamblers without a VSE (i.e., matched pairs). By subtracting the difference between the gamblers who opted for a VSE and their matched pairs (yielding the “difference-in-differences”), potential changes that might have occurred even without a VSE were separated out. The associated regression model was defined as follows:

$$log(stake) \sim {\beta _0} + {\beta _1}period + {\beta _2}intervention + {\beta _3}period:intervention$$

*Formula 1: Regression formula for measuring the relative changes in amount wagered after a VSE.*

The binary variable *period* indicates whether the stake was placed before or after the VSE, and the binary variable *intervention* indicates whether a player opted for a VSE or whether it was a matched pair without a VSE. Finally, *period:intervention* represents the interaction of the two variables.

Shapiro-Wilk tests (Shapiro et al., 1968) were performed to test for normality. Age (*S* = 0.96, *p* < .001) and amount of money wagered (*S* = 0.13, *p* < .001) significantly deviated from a normal distribution. Consequently, non-parametric tests were chosen for group comparisons. For the regression model in RQ2, the stepwise backward elimination with a threshold of *p* < .1 was applied (Sutter et al., 1993).

### Ethics

This study was performed in line with the principles of the Declaration of Helsinki and was approved by the last author’s university ethics committee.

## Results

### Gamblers returning from a VSE

Table 2 shows that 1,382 of 3,203 players in total (43.1%) started to gamble again after a VSE. More specifically, out of the 1,820 players who opted for a short-term VSE, 1,370 players (75.3%) started to gamble again during the study period. However, out of the 1,383 players who opted for a long-term VSE, only 12 players (0.9%) started to gamble again during the study period. The respective chi-square test showed that there was significant difference in the percentage of players returning from short-term and long-term VSEs (*χ*^2^ = 1,773.62, *p* < .001).

**Table 2:** Percentage of gamblers who returned after a VSE. There was no significant difference in the number of days until gamblers returned between short-term and long-term self-excluders (*U* = 7,678, *p* = .69)

VSE duration	Number of gamblers	Number of gamblers who returned	Median number of days until return
Short-term	1,820	1,370 (75.3%)	7
Long-term	1,383	12 (0.9%)	7
	3,203	43.1% (1,382)	7

To determine whether returning from a VSE was associated with previous amount of money wagered, the players who opted for a short-term VSE were split into four groups with respect to the amount of money wagered before the VSE. The respective number and percentage of players which started to gamble again were 291 (71.6%), 353 (76.6%), 363 (76.2%) and 363 (76.2%), with the first group comprising the lowest-stakes players and the last group comprising the highest-stakes players. The percentages of gamblers who returned were not significantly different between the four groups among short-term VSEs (*χ*^2^ = 3.29, *p* = .30). As only 12 gamblers returned from a long-term VSE, the authors concluded that this number was too low to further split long-term VSEs into four groups and perform a statistically valid comparison.

Table 3 reports the coefficients of the logistic regression including their *p*-values and confidence intervals (CIs). It shows that the variables significantly associated with increased odds to start gambling again after a VSE were (i) being female, (ii) having higher number of bets, (iii) having higher number of gambling days, (iv) having lower number of deposits per day, (v) having a shorter duration of the VSE, and (vi) playing fewer type of games.

**Table 3:** Coefficients of the logistic regression showing the behavioral variables, which were associated with a change in odds to start gambling again after a VSE.

Variable	*β*	*e*^*β*^-1	Std. error	*p*-value
Intercept	1.910***	-	0.283	< 0.001
Gender[male]	-0.430***	-	0.114	< 0.001
log(money wagered)	-0.086	-0.082	0.050	0.085
log(number of bets)	0.178**	0.195	0.054	0.001
Number of game types	-0.176*	-	0.073	0.016
Number of days	0.038**	-	0.013	0.004
Average number of deposits per day	-0.090**	-	0.028	0.001
VSE duration (weeks)	-0.252***	-	0.013	< 0.001
Pseudo *R*^2^ (CS)	0.513			
Number of observations	3,203			

Cox and Snell (CS); ***p < .001, **p < .01, *p < .05

### Gambling behavior after returning from a VSE

To evaluate the impact of a VSE on the money wagered after players returned to gambling, a matched control group was created by using a matched-pairs design. Therefore, the amount of money bet by gamblers who returned from a VSE could be compared to the amount of money bet by gamblers who did not opt for a VSE but had similar demographics and gambling behavior.

#### Validity of the matched control group

For 810 out of the 1,820 short-term self-excluders (44.5%), and for 603 out of the 1,383 long-term self-excluders (43.6%), a matched pair could be found. Out of the total group of short-term self-excluders 1,370 returned after a VSE (75.3%), whereas out of the 810 remaining players after the matching, 604 returned after a VSE (74.6%). There was no significant difference with respect to the number of players returning from a VSE between the entire sample and the remaining players after the matching (75.3% vs. 74.6%; *t*=-0.39, *p* = .70). Similarly, out of the total group of long-term self-excluders 12 returned from a VSE (0.9%), whereas out of the 603 remaining players after the matching, five returned from a VSE (0.8%). The respective statistical test reported no significant difference with respect to the number of players returning from a VSE between the entire sample and the remaining players after the matching (0.9% vs. 0.8%; *t*=-0.09, *p* = .93). Therefore, it can be concluded that the remaining players after matching did not significantly deviate from the entire sample, with respect to the probability of returning from the VSE. Furthermore, there was no significant difference in the amount of money wagered 30 prior the VSEs, compared to the matched pairs who did not opt for a VSE (*U* = 997,059, *p* = .477).

#### Impact of VSE on money wagered

After short-term VSEs, 243 gamblers wagered more than their respective matched pair (52.1%). A Z-test against the expected value of 0.5 (50%) was not significant (*z* = 0.83, *p* = .40). In other words, short-term self-excluders did not wager significantly less or more than their respective matched pairs after returning from a VSE. As only five long-term self-excluders returned from a VSE, the authors concluded that this number was too low for a statistically valid comparison with their respective matched pairs.

To measure the relative change in money wagered after the VSE compared to before the VSE, a difference-in-differences regression was used. Table 4 shows that both gamblers who opted for a VSE, as well as their matched pairs reduced their gambling over time (only 44.4% and 38.3%, respectively, gambled more than before). The difference-in-differences regression separated this concurrent reduction in wagering from the actual impact of the VSE on subsequent wagering. Table 5 reports the coefficients of the difference-in-differences regression. First, the coefficient on the *intervention* variable was not significant, which indicated that there was no significant difference in terms of money wagered between self-excluded players and their matched pairs before the VSEs. This was to be expected because the gamblers were matched on their gambling behavior and demographics. Second, the coefficient for the variable *period* was negative and significant, which means that the amount of money wagered decreased significantly over time for both the control group (i.e., the matched pairs) and the intervention group (i.e., players who opted for a VSE). Third, the non-significant coefficient on the interaction of the two variables suggested that the VSE did not have a significant effect on the wagers of players who returned from a VSE. This is in line with the previous finding, which showed that 52.1% of the players who opted for a short-term VSEs wagered more money after they returned, compared to their matched pairs. However, the result was not significantly different from the expected percentage of 50%. Therefore, the decrease in wagering after a short-term VSE was not caused by the VSE, but by a general trend that was also observed among the matched pairs who did not opt for a VSE.

**Table 4:** Gambling behavior of individuals who opted for a VSE, compared to their matched pair (MP)

VSE duration	Number of gamblers	Number of gamblers who return		Gamble more than MP	Gamble more than before	
		VSE	MP		VSE	MP
Short-term	810	604 (74.6%)	622 (76.8%)	243 (52.1%)	268 (44.4%)	238 (38.3%)
Long-term	603	5 (0.8%)	289 (47.9%)	3 (75.0%)	3 (60.0%)	126 (43.6%)
	1,413	609 (43.1%)	911 (64.5%)	246 (52.1%)	271 (44.5%)	364 (40.0%)

Note: Only 5 out of 603 (0.8%) gamblers returned after a long VSE.

**Table 5:** Difference-in-differences regression to measure the relative change in money wagered after gamblers returned from a VSE.

Variable	*β*	Std. error	*p*-value	CI
Intercept	6.791***	0.081	< 0.001	[6.632, 6.950]
Intervention	0.002	0.115	0.983	[-0.223, 0.227]
Period	-0.606***	0.115	< 0.001	[-0.831, -0381]
Intervention:period	0.096	0.162	0.555	[-0.222, 0.414]
*R* ^2^	0.025			

***p < .001, **p < .01, *p < .05

## Discussion

The present study analyzed the gambling behavior of a secondary dataset comprising 3,203 British online gamblers who voluntarily self-excluded between January 2021 and August 2022. The study’s goal was to investigate the likelihood of returning from a VSE and changes in the amount of money spent after returning from a VSE compared to the behavior preceding a VSE. Given that players could not be randomly assigned to different experimental conditions, a matched-pairs design was chosen to obtain a control group and control for seasonal effects. On average, gamblers were 39 years old and 51.3% of the sample were male. Based on the distribution of the duration of the VSE (Fig. 1), the authors distinguished between short-term VSEs (up to 38 days) and long-term VSEs (90 days or longer). The most frequent VSE durations were 30, 90 and 180 days. There was no significant difference with respect to age between players who opted for a short-term VSE and a long-term VSE. Players who opted for a short-term VSE were significantly more likely to be female, wagered significantly more money in the 30 days prior to the VSE, and gambled significantly more often in the same period compared to gamblers who opted for a long-term VSE.

### Efficacy of VSE to stop gambling (RQ1)

A quarter of players who opted for a short-term VSE did not return to gamble and more than 99% of players who opted for a long-term VSE did not return to gamble. The higher percentage of gamblers who returned after a short-term VSE may indicate lower efficacy of short-term VSE compared to long-term VSE, which is supported by a previous study that reported no significant short-term effects on gambling behavior after VSEs (Caillon et al., 2019). Despite these large differences, the results should be interpreted with caution due to the limited study period. Players with a long-term VSE may have returned to gamble further in the future after the study period had ended. Although the present study enabled each gambler a period of at least 30 days to return after the expiration of the VSE, players who opted for a long-term VSE have less time to return than short-term VSE because the study period ended in August 2022. However, when players returned from a VSE, there was no significant difference between the number of days it took gamblers to return (i.e., a median of seven days for both long-term and short-term VSEs – see Table 2), suggesting that the impact of the limited study period may have been negligible. Another reason why most gamblers did not return after a VSE might be that they switched to other gambling operators because a survey among only gamblers reported that 38% said they gambled with other operators during their VSE (Håkansson & Widinghoff, 2020). In addition, not returning from a long-term VSE may be associated with a high rate of spontaneous remission from a gambling disorder (Fröberg, 2015).

### Risk factors for a return to gambling after a VSE (RQ2)

In the present study, the gender ratio for self-excluders was found to be approximately equal, which is in line with previous research (Nower & Blaszczynski, 2006). However, the present results suggest that males were less likely to return to gamble from a VSE than females. To the best of the authors’ knowledge, no previous study has examined the role of gender in the efficacy of VSE. Another explanation why males are less likely than females to return to gambling could be that they might be more likely to switch to other gambling platforms to circumvent their VSE.

Surprisingly, high-spending gamblers did not return to gamble more often from a short-term VSE than low or average spending players. This meant that the likelihood of starting gambling again after a short-term VSE was not associated with the amount of money spent prior to a VSE. This was perhaps unexpected because one recent study found that gamblers who were the most heavily involved in terms of the amount of money wagered showed no effects after a short-term VSE, whereas the remaining gamblers showed a significant reduction in the amount of time and money spent after a short-term VSE (Luqiens et al., 2019). However, the results of the present study showed that the number of bets and number of gambling days were positively associated with the likelihood of starting to gamble again after a VSE. This finding suggests that the more time individuals spend gambling, the more difficult it is for them to stop gambling. Further research is needed to better understand what behavioral markers might identify individuals at-risk for returning to gambling after a VSE, which in turn can be used to develop more personalized recommendations to support gamblers if they do return, such as the operators’ promoting stricter time limits on gambling.

### Changes in wagering due to a VSE (RQ3)

The result of the difference-in-differences regression suggested that there was no significant decrease or increase in the amount of money wagered after gamblers returned from a VSE compared to the matched control group. Therefore, short-term VSEs did not have a significant effect on gamblers’ wagering once they returned. This result is surprising in relation to a previous study using player-tracking data which reported that the amount of money wagered significantly decreased after a VSE compared to a matched control group (Luqiens et al., 2019). There could be several reasons for the different results. First, the study by Luqiens et al. (2019) included online poker players, whereas the present study comprised online casino players. Second, the previous study formed a control group by matching players on age, account age, and gender, whereas the present study matched players on their age, gender, wagering, number of active days, and game type profile. Third, the previous study analyzed gambling behavior after the expiration of a VSE, whereas the present study used the point in time when players started gambling again to ensure a comparable observation period for all players. Given the limited number of previous studies, these findings warrant further investigation to isolate potentially confounding factors, and further evaluate whether returning after a VSE should be considered as risk factor, given that gamblers may not have changed their problematic gambling behavior.

To reduce the risk of problematic gambling behavior after returning from a VSE, operators could require gamblers to conduct an online self-test on problem gambling (Jonsson et al., 2017). This might facilitate the identification of potentially problematic gambling behavior, allowing the operator to intervene to prevent further harm. By making such self-tests mandatory, gamblers might be encouraged to reflect on their gambling behavior and seek help if necessary, and therefore contributing to a more responsible gambling environment. In addition, operators should offer additional support or resources, such as referral to counseling programs, to gamblers who show signs of problem gambling after returning from a VSE to help them control their gambling behavior. Finally, regulators should promote initiatives that prevent gamblers from circumventing their VSE by gambling on other legal gambling platforms. For instance, in the UK, the Gambling Commission have set the British online gambling operators an *“industry challenge”* to explore a *“single customer view”* where a gambler who voluntarily self-excludes from one online gambling site is excluded from all other sites through the sharing of customer data between online gambling operators (Gambling Commission, 2021).

### Limitations

Due to the limited time period of the present study (i.e., from January 2021 until August 2022), it cannot be ruled out that individuals started to gamble again after the study period. Moreover, gamblers might have switched to another gambling operator to bypass their VSE. However, the study only included individuals whose VSE ended at least 30 days before the end of the study period, and out of all gamblers who returned, 50% started to gamble again within seven days after the end of their VSE. Another limitation is the lack of information about the individuals’ gambling severity and their motivations for VSE. There might be differences in the efficacy of short- and long-term VSE, depending on the individuals’ gambling severity and their motivation to self-exclude. Furthermore, it was not possible to assign players to experimental groups at random because opting for a self-exclusion is a voluntary behavior. Instead, a natural experiment was conducted by creating a matched-control group. Finally, the study comprised only online casino players from one gambling operator. Further research is needed among different gambling populations and gambling operators to better understand the impact of VSE on online gambling behavior among affected individuals.

## Conclusion

Voluntary self-exclusion is a mandatory responsible gambling instrument in most regulated online gambling markets. However, there has been limited research on the efficacy of online VSEs. The present study showed that most individuals who opted for a short-term VSEs returned to gamble during the study period. In contrast, only a minority of individuals started to gamble again after a long-term VSE. The results suggested that short-term VSEs had no significant impact on the amount wagered after gamblers started to gamble again. As only a small number of gamblers returned from a long-term VSE it was not possible to statistically evaluate whether gamblers changed their wagering after starting to gamble again. Therefore, long-term VSE might be more effective because most gamblers did not return to gambling. Overall, online gambling operators should monitor players who return from a VSE, especially if their self-exclusion was due to problem gambling because the present results indicated that gamblers did not change their behavior after a VSE.

## Data Availability

Not applicable.

## Appendix 1

**Table:** List of all variables used to measure the gambling behavior (all values calculated using the last 30 days prior to a VSE).

Variable	Description
Gender	Gender of the gamblers (defaults to female)
Age	Age of the gamblers (in years)
Money wagered	Total amount of money wagered
Money deposited	Total amount of money deposited
Percentage loss	Amount of money lost divided by money wagered
Number of deposits	Total number of deposits
SD amount deposits	Standard deviation of the amount of money deposited
Number of withdrawals	Total number of withdrawals
SD amount withdrawals	Standard deviation of the amount of money withdrawn
Number of cancelled withdrawals	Total number of cancelled withdrawals
Number of bets	Total number of bets
Number of games	Total number of different games played
Number of game types	Total number of different game types played
Number of payment methods	Total number of different payment methods used
Number of days	Total number of active gambling days
Average number of deposits per day	Average number of deposits per day
Average number of bets per day	Average number of bets per day
Break duration	The duration of the VSE (in weeks)
